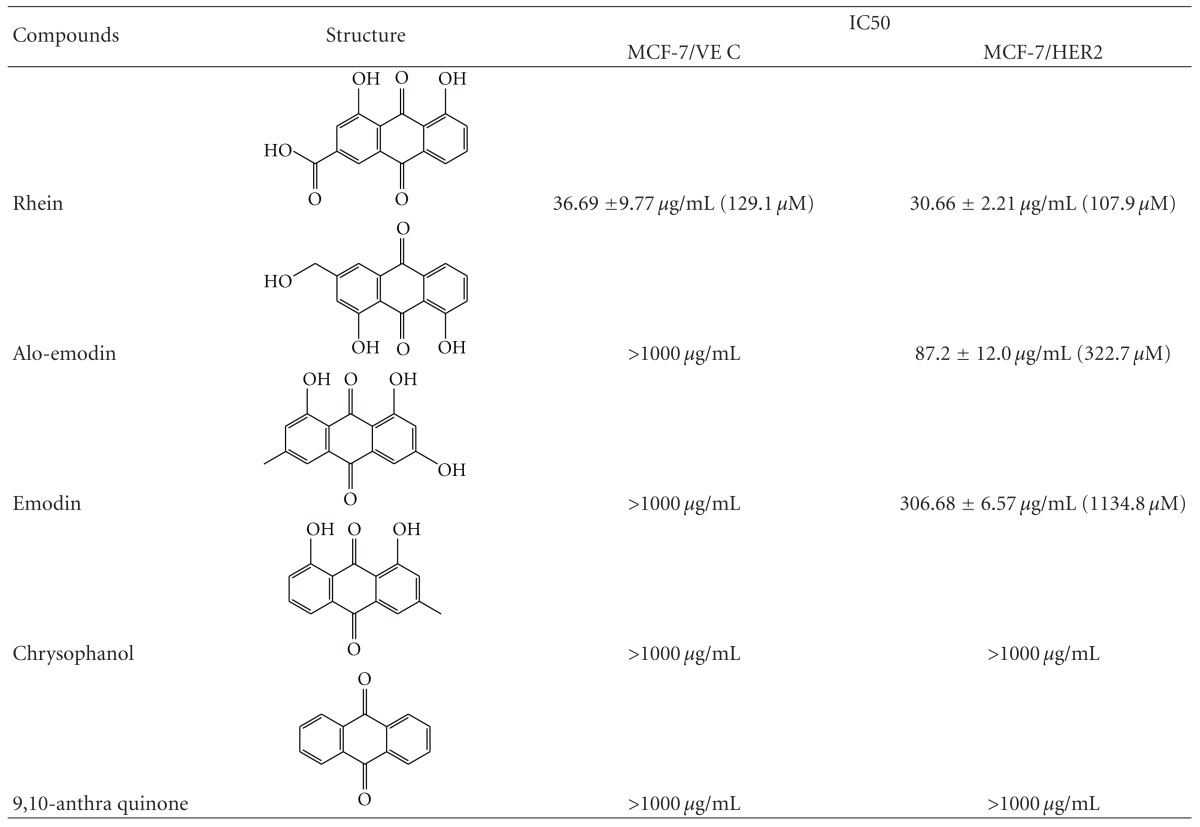# Rhein Induces Apoptosis in Human Breast Cancer Cells

**DOI:** 10.1155/2012/952504

**Published:** 2011-10-05

**Authors:** Ching-Yao Chang, Hong-Lin Chan, Hui-Yi Lin, Tzong-Der Way, Ming-Ching Kao, Ming-Zhang Song, Ying-Ju Lin, Cheng-Wen Lin

**Affiliations:** ^1^Department of Biotechnology, Asia University, Taichung 413, Taiwan; ^2^Institute of Bioinformatics and Structural Biology, Department of Medical Science, National Tsing Hua University, Hsinchu 300, Taiwan; ^3^School of Pharmacy, China Medical University, Taichung 404, Taiwan; ^4^Department of Biological Science and Technology, China Medical University, Taichung 404, Taiwan; ^5^Department of Medical Laboratory Science and Biotechnology, China Medical University, Taichung 404, Taiwan; ^6^Department of Medical Genetics and Medical Research, China Medical University Hospital, Taichung 404, Taiwan

## Abstract

Human breast cancers cells overexpressing HER2/*neu* are more aggressive tumors with poor prognosis, and resistance to chemotherapy. This study investigates antiproliferation effects of anthraquinone derivatives of rhubarb root on human breast cancer cells. Of 7 anthraquinone derivatives, only rhein showed antiproliferative and apoptotic effects on both HER2-overexpressing MCF-7 (MCF-7/HER2) and control vector MCF-7 (MCF-7/VEC) cells. Rhein induced dose- and time-dependent manners increase in caspase-9-mediated apoptosis correlating with activation of ROS-mediated activation of NF-**κ**B- and p53-signaling pathways in both cell types. Therefore, this study highlighted rhein as processing anti-proliferative activity against HER2 overexpression or HER2-basal expression in breast cancer cells and playing important roles in apoptotic induction of human breast cancer cells.

## 1. Introduction

Rhubarb root (Rheum palmatum), member of the Polygonaceae family, is one well-known antineoplastic herb in traditional Chinese medicine [1]. Anthraquinone derivatives are its main bioactive constituents: emodin (1,3,8-trihydroxy-6-methylanthraquinone), aloe emodin (1,8-dihydroxy-3-hydroxyl-methyl anthraquinone), rhein (1,8-dihydroxy-3-carboxyanthraquinone), chrysophanol (1,8-dihydroxy-3-methyl-anthraquinone), physcion (1,8-drihydroxy-3-methyl-6-methoxyanthraquinone), and danthron (1,8-dihydroxy-9,10-anthraquinone). Rhubarb anthraquinone derivatives can induce apoptosis of human cancer, including lung adenocarcinoma A549, myelogenous leukemia HL-60, lung squamous carcinoma CH27, cervical carcinoma HeLa cells, neuroblastoma IMR-32, bladder cancer T24, and hepatoma HepG2 cells [1]. Emodin inhibits cellular proliferation, induces apoptosis, and prevents metastasis through activation of tyrosine kinases, phosphoinositol 3-kinase (PI3K), protein kinase C (PKC), NF-kappa B (NF-*κ*B), and mitogen-activated protein kinase (MAPK) signaling cascades [2–5]. Aloe-emodin has antitumor properties through the p53 and its downstream p21 pathway [6]. Emodin and aloe emodin also reduce tumor size, prolong survival, decrease incidence of tumor invasion and neovascularization using *in vivo* animal models [7, 8]. Rhein blocks the uptake of glucose in tumor cells, causing changes in membrane-associated functions to trigger cell death [9].

Breast cancer is one of the most common cancers in women, resulting from gene amplification and/or overexpression of some oncogenes like HER2/*neu* (also known as ErbB2) and oestrogen receptors [10, 11]. Overexpression of HER2/*neu* in nearly 30% of human breast cancers correlates with more aggressive tumors and poor prognosis [12]. HER-2-overexpressing cells appear to be resistant to some classes of chemotherapy agents [12], but sensitive to others [13]. Overexpression of HER2/*neu* was positively correlated with p53 nuclear accumulation and tumor metastasis, negatively with hormonal receptor status [14]. Tyrosine kinase activity of HER2/*neu* could phosphorylate HER3 and then activate phosphatidylinositol 3-kinase (PI3K) involved in malignance of tumors [15]. Trastuzumab (Herceptin) is widely used for treatment of HER2-positive breast cancer; resistance to trastuzumab occurs in some patients [16]. Therefore, HER2/*neu* is a potentially therapeutic target for breast cancer, influencing efficacy of chemotherapy.

Engineering human breast cancer MCF-7 cells that expresses basal level of HER2/*neu *for Overexpression of HER2/*neu* has been used to analyze biological properties of HER2 overexpression [15, 17]. This study investigates apoptotic effects of anthraquinone derivatives of rhubarb root on both types of breast cancer cells, HER2-overexpressing MCF-7 cells (MCF-7/HER2) and control vector MCF-7 cells (MCF-7/VEC). Of 7 anthraquinone derivatives, only rhein showed antiproliferative and apoptotic effects on both MCF-7/HER2 and MCF-7/VEC cells. We also analyzed apoptotic mechanism of rhein on both human breast cancer cell lines with basal level and Overexpression of HER2. 

## 2. Materials and Methods

### 2.1. Cell Culture

Human breast cancer cell lines, vector control MCF-7 cells (MCF-7/VEC) and HER2-overexpressing MCF-7 cells (MCF-7/HER2), were used in this study, as previously described [15]. Both types were grown in DMEM/F-12 (Invitrogen) with 10% fetal bovine serum (Invitrogen), gentamicin (50 mg/mL) and G418 (800 *μ*g/mL).

### 2.2. Western Blotting Analysis

Cell lysates that were harvested from MCF-7/VEC and MCF-7/HER2 cells with or without treatment of anthraquinone derivatives were dissolved in 2X SDS-PAGE sample buffer without 2-mercaptoethanol, and boiled for 10 min. Cell lysate proteins were resolved on 12% SDS-PAGE gels and transferred to nitrocellulose paper. Resultant blots were blocked with 5% skim milk and reacted with properly diluted monoclonal antibodies against HER2/c-*neu* (Ab-3,3B5), caspase 9, Apoptosis signal-regulating kinase 1 (ASK1), P21, and *β*-actin (Cell Signaling Technology) for 3 h incubation. Immune complexes were detected by horseradish peroxidase-conjugated goat anti-mouse IgG antibodies, followed by enhanced chemiluminescence reaction (Amersham Pharmacia Biotech).

### 2.3. MTT Cytotoxicity Test

Aloe emodin, emodin, rhein, chrysophanol, sennoside A, sennoside B, and 9-, 10-anthraquinone were purchased from Sigma Chemical Company (St. Louis, MO, USA).* In vitro* antiproliferative effect of each compound on MCF-7/VEC and MCF-7/HER2 cells was examined using MTT assay. Cells were plated in 96-well plates (5 × 10^4^ cells/well) and then treated with serial dilution of each tested compound. After treatment for 48 h, 25 *μ*L of a MTT solution at 5 mg/mL was added to each well and incubated at 37°C in 5% CO_2_ for 3 h. After subsequent washing three times with phosphate buffer saline, 100 *μ*L DMSO was added into plates for dissolving formazan crystals. OD_570-630_ in each well was measured with a micro-ELISA reader and survival rate used to indicate suppressive effects of each compound on MCF-7/VEC and MCF-7/HER2 cells. Survival rate (%) = ((A_control_ − A_experiment_)/A_control_) × 100%. Cytotoxic concentration giving 50% (CC50) was calculated by computer program (provided by John Spouge, NCBI, NIH).

### 2.4. Detection of Cell Cycle and Apoptosis by Flow Cytometry

MCF-7/VEC and MCF-7/HER2 cells were harvested 24,48, and 72 h posttreatment with or without serial dilutions of rhein. Cells were fixed using 70% ethanol at 4°C overnight, then re-suspended in PBS containing 50 *μ*g/mL PI and 0.1 mg/mL RNase and 0.1% Triton X-100 in darkroom. After 30 min incubation at 37°C, cells were tested by flow cytometry (Becton-Dickinson, San Jose, Calif, USA) equipped with an argon ion laser at 488 nm wavelength, then rates of sub G1 (apoptotic), G1, S, and G2 phase cells were determined. In addition, rhein-induced cytopathic effect (CPE) of MCF-7/VEC and MCF-7/HER2 cells was observed 48 h posttreatment using light microscopy.

### 2.5. Detecting Intracellular Reactive Oxygen Species (ROS) by Flow Cytometry

MCF-7/VEC and MCF-7/HER2 cells were harvested 24 h posttreatment with or without serial dilutions of rhein; 5 × 10^5^ cells were harvested after 48 h transfection and their ROS level noted. They were washed twice with PBS, then incubated with 10 *μ*M 2,7-dichlorodihydrofluorescein diacetate (DCFH-DA, Sigma) at 37°C for 30 min in darkroom for final analysis by flow cytometry (Becton Dickinson FACS Calibur).

### 2.6. In Vivo Signaling Transduction Pathway Assay with Cis-Reporter Plasmids

Cis-reporter plasmids pAP1- Luc, pNF-*κ*B-Luc, and p53-Luc were purchased from Stratagene Company. MCF-7/VEC and MCF-7/HER2 cells were co-transfected with cis-reporter plasmid and internal control reporter pRluc-C1 (BioSignal Packard) in 6-well plates, using the *Arrest-In* transfection reagent. After one-day incubation, transfected cells were seeded into 24-well plates using the MEM containing 10% FBS, then treated with or without 20 *μ*g/mL rhein. After 4 h posttreatment, enzyme activity of firefly and Renilla luciferases in indicated cells was measured by dual Luciferase Reporter Assay System (Promega) and Luminometer TROPIX TR-717 (Applied Biosystems). Relative firefly luciferase activity of the cis-acting reporter was normalized by Renilla luciferase.

### 2.7. Statistical Analysis

ANOVA analysis using SPSS program (version 10.1, SPSS Inc., IL, USA) or Student's *t*-test analyzed data, *P* value below 0.05 were considered statistically significant.

## 3. Results and Discussion

### 3.1. Rhein Inhibited Growth of MCF-7/VEC and MCF-7/HER2 Cells in Dose-Dependent Manner

**Figure 1:**
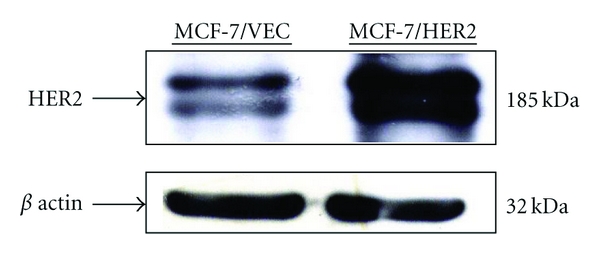
Expression of HER2/*neu* in human breast cancer cells. Vector control MCF-7 cells (MCF-7/VEC) and HER2-overexpressing MCF-7 cells (MCF-7/HER2) were harvested, and cell lysates were analyzed by Western blotting with anti-HER2 and anti-*β* actin antibodies.

**Figure 2:**
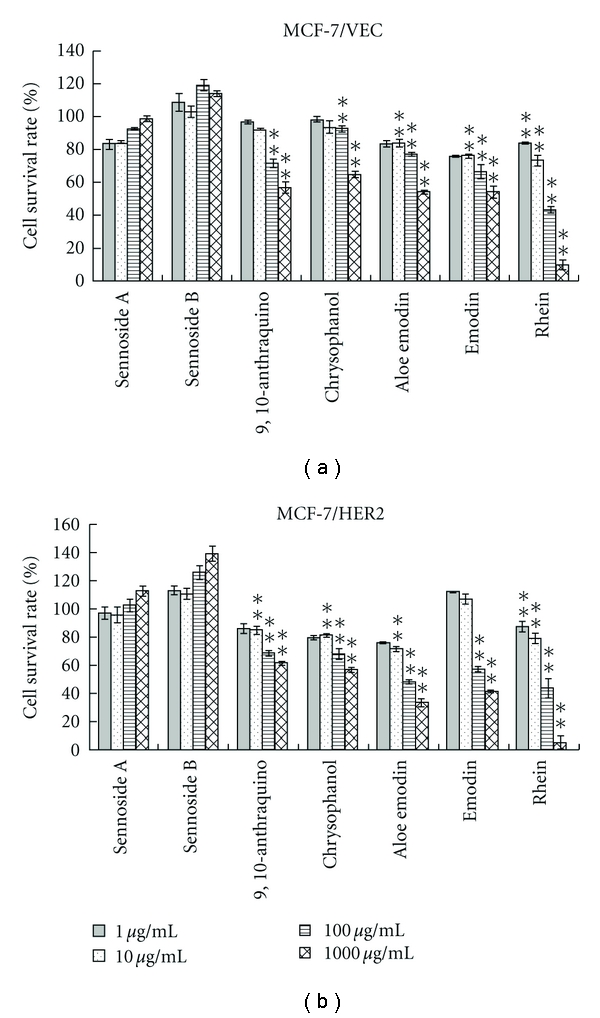
Effect of anthraquinone derivatives on the growth of human breast cancer cells. MCF-7/VEC (a) and MCF-7/HER2 (b) cells were plated in 96-well plates (5 × 10^4^ cells/well) and then treated with serial dilution of each tested compound. After treatment for 48 h, cell growth was examined using MTT assay. OD_570-630_ in each well was measured with a micro-ELISA reader. Survival rate (%) = ((A_control_ − A_experiment_)/A_control_) × 100. *P  value < 0.05; **P  value < 0.01 compared with untreated cells.

 To examine expression level of HER2 in MCF-7/VEC and MCF-7/HER2 cells, both cell lysates were analyzed using Western blotting assay. Immunoreactive bands with anti-HER2 antibodies revealed basal expression of HER2 in MCF-7/VEC cells and Overexpression of HER2 in MCF-7/HER2 cells (Figure 1, Lanes 1 and 2). Subsequently, antiproliferative effect of anthraquinone derivatives on MCF-7/VEC and MCF-7/HER2 cells was analyzed using MTT assay (Figure 2). Sennosides A and B have no inhibitory effect on cell growth, while IC50 values of 9-, 10-anthraquinone, chrysophanol, aloe emodin, and emodin for both types of cells exceeded 100 *μ*g/mL (Table 1). Only rhein had a dose-dependent antiproliferative ability on both types. In addition, rhein was less toxic to nontumorigenic MCF-10A cells used as the normal control (Figure  1 of the Supplementary material available online at doi:10.1155/2012/940920). The IC50 value of rhein was 36.69 ± 9.77 *μ*g/mL (129.1 ± 34.37 *μ*M) for MCF-7/VEC cells and 30.66 ± 2.21 *μ*g/mL (107.9 ± 7.7 *μ*M) for MCF-7/HER2 cells. Rhein showed micromolar IC50 values on growth suppression of MCF-7/VEC and MCF-7/HER2 cells, similar to antiproliferative activity of rhein lysinate on human breast cancer cell lines MCF-7, SK-Br-3, and MDA-MB-231 [18] and human hepatocellular carcinoma BEL-7402 cells [19], but less potent than its activity against tongue cancer cell line SCC-4 [20] and ovarian carcinoma cell line SKOV-3 [21].

### 3.2. Rhein Influenced Cell Cycle Phase of MCF-7/VEC and MCF-7/HER2 Cells

**Figure 3:**
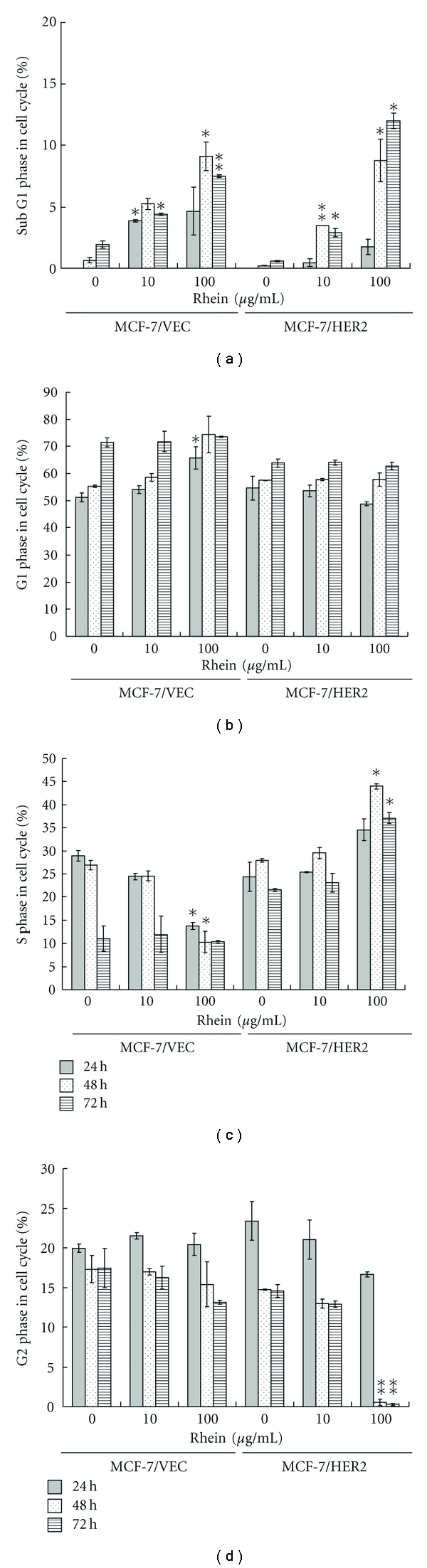
Cell cycle analysis of human breast cancer cells in response to rhein. MCF-7/VEC and MCF-7/HER2 cells were treated with serial dilution of rhein. After 24, 48, and 72 h incubation, cells were fixed by 70% ethanol, stained with PI, and analyzed using flow cytometry. Percentage of cells in sub G1 (apoptotic) (a), G1 (b), S (c), and G2 (d) phases were shown representing three independent studies. *P  value < 0.05; **P  value < 0.01 compared with untreated cells.

**Figure 4:**
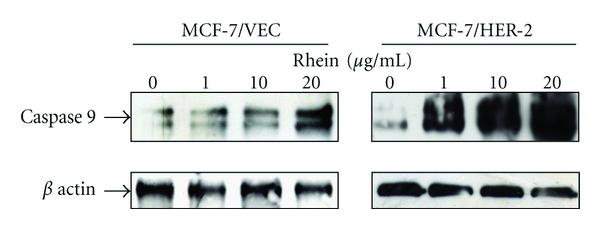
Western blotting analysis of caspase 9 in human breast cancer cells with/without rhein treatment. After 48 h incubation with rhein, MCF-7/VEC and MCF-7/HER2 cells were harvested, and lysates were analyzed by Western blotting with anticaspase-9 and anti-*β* actin antibodies.

To examine the effect of rhein on cell cycle phases, both types were treated with or without rhein at concentrations of 10 *μ*g/mL and 100 *μ*g/mL, and then harvested 24, 48, and 72 hours posttreatment. Cells were stained with propidium iodide and analyzed for cell cycle fractions by flow cytometry (Figure 3). Rhein induced concentration- and time-dependent manner increase in sub-G1 (apoptosis) fractions of MCF-7/VEC and MCF-7/HER2 cells (*P* < 0.05, Figure 3(a)). The rate of G1 phase in MCF-7/VEC cells significantly increased 24 hours posttreatment with rhein at a concentration of 100 *μ*g/mL (*P* < 0.05), but no effect of rhein on G1 phase in MCF-7/HER2 cells was detected (Figure 3(b)). Rhein at concentrations of 10 and 100 *μ*g/mL caused a significant S phase arrest in MCF-7/HER2 cells 48 and 72 hours posttreatment (*P* < 0.05), but rhein at a concentration of 100 *μ*g/mL decreased S phase fractions in MCF-7/VEC cells 24 and 48 hours posttreatment (*P* < 0.05, Figure 3(c)). Interestingly, a very low fraction of G2 phase was detected in MCF-7/HER2 cells 48 and 72 hours posttreatment with rhein at a concentration of 100 *μ*g/mL (*P* < 0.05); no significant change appeared in MCF-7/VEC cells 24, 48, and 72 hours posttreatment (Figure 3(d)). Cell cycle analysis demonstrated rhein inducing S phase arrest and G2 phase decrease in MCF-7/HER2 cells (Figures 3(c) and 3(d)). Moreover, rhein increased the rate of G1 phase, but decreased the fraction of S phase in MCF-7/VEC cells (Figures 3(b) and 3(c)). The difference of cell cycle distribution in response to rhein could be due to cell specificity [22, 23]. Cell cycle analysis of breast cancer BT-474 cells and MCF-7 cells indicated 1alpha(OH)D5 inducing an S phase decrease in MCF-7 cells and a G2 phase decrease in BT-474 cells [22]. Moreover, resveratrol induced apoptosis of MCF-7 cells via S phase arrest, but not MDA-MB-231 cells [23]. Western blot analysis indicated activation of caspase 9 in both cell types (Figure 4), responsible for dose-dependent manners on rhein-induced apoptosis of breast cancer cells. Results demonstrate that S phase arrest of MCF-7/HER2 cells and increased G1 phase of MCF-7/VEC cells correlated with rhein-induced caspase-9-mediated apoptosis. Rhein-induced S phase arrest of MCF-7/HER2 cells correlates with antiproliferative and apoptotic mechanisms of rhein on human hepatocellular and tongue carcinoma cells [19, 24]. Also, G1 arrest of MCF-7/VEC cells induced by rhein proves consistent with rhein-induced apoptosis of human lung cancer A-549 cells [25] and cervical cancer Ca Ski cells [26].

### 3.3. Rhein Induced ROS-Mediated Activation of ASK1 in MCF-7/VEC and MCF-7/HER2 Cells

**Figure 5:**
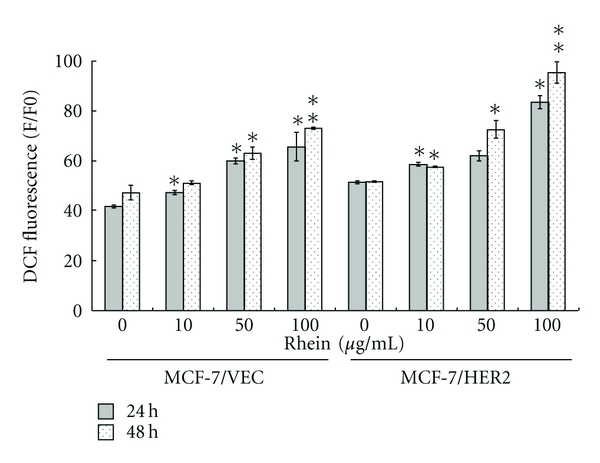
Flow cytometric analysis of reactive oxygen species (ROS) in human breast cancer cells with/without rhein treatment. After 24 and 48 h incubation with rhein, MCF-7/VEC and MCF-7/HER2 cells were harvested, and then stained by DCFH-DA dye. The fluorescence intensity of stained cells was determined by flow cytometry. *P  value < 0.05; **P  value < 0.01 compared with untreated cells.

**Figure 6:**
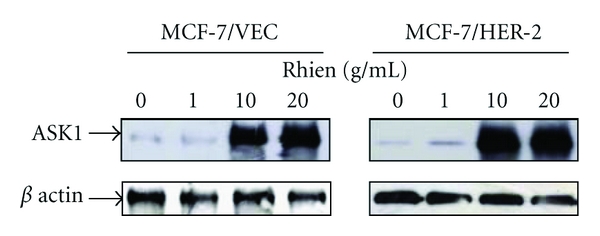
Western blotting analysis of ASK1 expression in human breast cancer cells with/without rhein treatment. After 48 h incubation with rhein, MCF-7/VEC and MCF-7/HER2 cells were harvested, and lysates were analyzed by Western blotting with anti-ASK1 and anti-*β* actin antibodies.

Intracellular ROS generation reportedly triggers activation of caspase 9 and ASK1 signaling in apoptotic responses [27–29]. Therefore, we assessed effects of rhein treatment on intracellular ROS and ASK1 levels in MCF-7/VEC and MCF-7/HER2 cells (Figures 5 and 6). Cells were cultured with rhein at 37°C for 48 hours. After being washing twice with PBS, cells were stained with DCFH-DA, then analyzed by flow cytometry. Rhein treatment caused concentration-dependent increase of intracellular ROS in MCF-7/VEC and MCF-7/HER2 cells (*P* < 0.05, Figure 5). Western blot with anti-ASK1 antibodies revealed rhein as concentration-dependently triggering ASK1 level in each type of cell (Figure 6). Results indicate ROS-mediated ASK1 signaling in rhein-induced apoptosis of both types. The findings were in agreement with incremental level of intracellular ROS in rhein-induced apoptosis of human tongue cancer cells, promyelocytic leukemia cells, and nasopharyngeal carcinoma cells [24, 30, 31].

### 3.4. Rhein Activated p53/p21 Signaling in MCF-7/VEC and MCF-7/HER2 Cells

**Figure 7:**
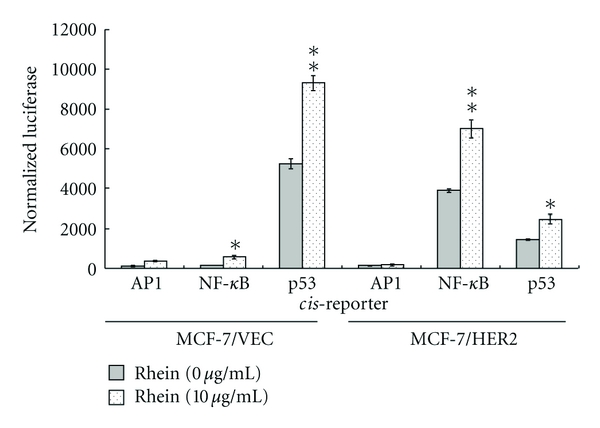
Effect of rhein on *in vivo* signal pathways in human breast cancer cells. MCF-7/VEC and MCF-7/HER2 cells were transiently co-transfected with cis-reporter plasmids (pAP1-Luc, pNF-*κ*B-Luc, and p53-Luc) and an internal control reporter (pRluc-C1), and then treated with rhein for 24 h. Firefly and Renilla Luciferase enzymes were measured; the relative firefly luciferase activity was normalized by Renilla Luciferase. *P  value < 0.05; **P  value < 0.01 compared with untreated cells.

**Figure 8:**
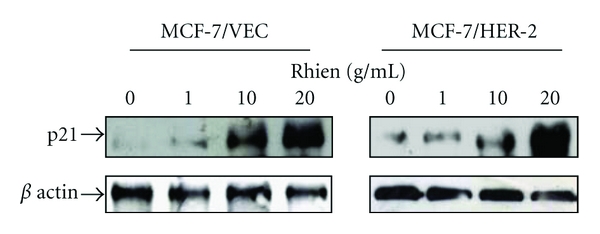
Western blotting analysis of p21 expression in human breast cancer cells with/without rhein treatment. After 48 h incubation with rhein, MCF-7/VEC and MCF-7/HER2 cells were harvested, and lysates were analyzed by Western blotting with anti-p21 and anti-*β* actin antibodies.

To probe correlation between *in vivo* signaling pathways and rhein-induced growth arrest and apoptosis, cells in response to rhein were further characterized by dual reporter assays with internal control reporter pRluc-C1 and luciferase reporter plasmid containing direct repeat elements of AP1, NF-*κ*B, and p53 (Figure 7). The cells were harvested 4 h posttreatment, and then the relative expression of firefly luciferase driven from the indicated cis-reporter plasmid was normalized by Renilla luciferase. Relative intensity of firefly luciferase revealed that rhein significantly activated p53- and NF-*κ*B promoters (*P* < 0.05), but induced no significant change of AP1-derived reporter activity in each type of cell (Figure 7). Moreover, p53- or NF-*κ*B-derived reporter activity increased more than 1.7-fold in rhein-treated cells than mock cells. Definite increase of rhein-induced NF-*κ*B- and p53-derived reporter activity in both types of cells indicated rhein modulating NF-*κ*B- and p53-signaling pathways in breast cancer cells. Our result was inconsistent with the function of NF-*κ*B mediating the cell survival response by inhibiting p53-dependent apoptosis and upregulating anti-apoptotic members of the Bcl2 family [32]. However, recent evidences show pro-apoptotic property of NF-*κ*B via p53-dependent apoptosis in breast cancer MCF7/ADR, melanoma M14, and pro-B cells [33–36]. Activation of NF-*κ*B induces upregulation of pro-apoptotic death receptor 5 (DR5) in breast cancer MCF-7 and MDA-MB-231 cells, correlating with TNF-related apoptosis inducing ligand-mediated apoptosis [37]. In addition, oxidative stress has demonstrated activation of both NF-*κ*B and p53 [38, 39]. Doxycycline-induced superoxide-mediated apoptosis indicated NF-*κ*B as a proapoptotic factor by activating the p53-signaling pathway [39]. Monoamine neurotoxin-induced apoptosis of peripheral blood lymphocytes is associated with activation of NF-*κ*B, p53, and c-Jun transcription factors [40]. Thus, rhein-induced activation of both NF-*κ*B- and p53-signaling pathways might correlate with ROS-mediated ASK1 signaling in breast cancer cells (Figures 5 and 6). Moreover, rhein has reported activation of p53/p21 signaling pathway with in rhein-induced apoptosis of human lung cancer cells, cervical cancer cells, and hepatoblastoma cells [25, 26, 41]. We also demonstrated rhein-inducing dose-dependent increases of p21 protein in MCF-7/VEC and MCF-7/HER2 cells (Figure 8). Therefore, rhein-induced apoptosis of breast cancer cells could be associated with ROS-mediated activation of NF-*κ*B- and p53-signaling pathways.

In conclusion, this study highlighted rhein versus six other anthraquinone derivatives, as processing antiproliferative activity against both types of breast cancer cells with HER2 overexpression or HER2-basal expression. Rhein showed micromolar IC50 values on growth inhibition of both types via caspase-9-mediated apoptosis. ROS-mediated activation of NF-*κ*B- and p53-signaling pathways might play important roles in rhein-induced apoptosis of human breast cancer cells.

##  Conflict of Interests

All authors report no conflict of interests relevant to this paper.

## Supplementary Material

Supplemental Figure 1: To compare cytotoxic effect of rhein on normal breast MCF-10A cells, and breast cancer MCF-7/VEC and MCF-7/HER2 cells, the cells were treated with 20 **μ**g/mL rhein for 48 h. The cell survival rate was determined using MTT assay, showing 99.7 ± 0.4% for MCF-10A cells, 75.5 ± 3.5% for MCF-7/VEC cells, and 59.1 ± 1.4% for MCF-7/HER2 cells, respectively. The resulted demonstrated rhein being less toxic to non-tumorigenic MCF-10A cells.

## Figures and Tables

**Table 1:** IC50 values of anthraquinone derivatives in MCF-7/VEC and MCF-7/HER2 cells.