# Supplementary material for: Rhein Induces Apoptosis in Human Breast Cancer Cells
Source: Evid Based Complement Alternat Med. 2011 Oct 5;2012:952504. doi: 10.1155/2012/952504 (PMC3189565; doi:10.1155/2012/952504)
Supplement: Supplementary file 1 — Supplemental Figure 1: To compare cytotoxic effect of rhein on normal breast MCF-10A cells, and breast cancer MCF-7/VEC and MCF-7/HER2 cells, the cells were treated with 20 μg/mL rhein for 48 h. The cell survival rate was determined using MTT assay, showing 99.7 ± 0.4% for MCF-10A cells, 75.5 ± 3.5% for MCF-7/VEC cells, and 59.1 ± 1.4% for MCF-7/HER2 cells, respectively. The resulted demonstrated rhein being less toxic to non-tumorigenic MCF-10A cells. [file 952504.f1.pdf]

Supplemental Figure

Supplemental Fig. 1.

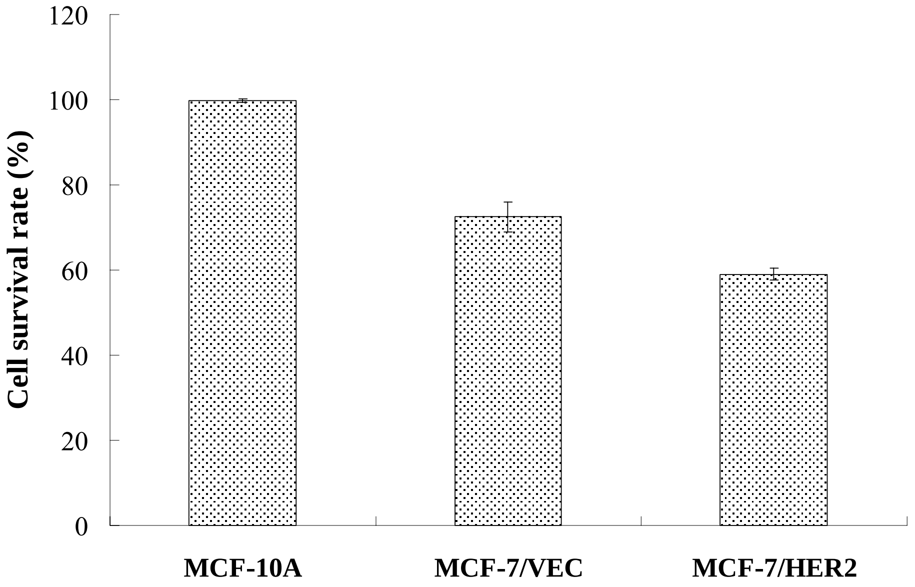

Supplemental Fig. 1. Cytotoxic effect of rhein on normal breast MCF-10A cells, and breast cancer MCF-7/VEC and MCF-7/HER2 cells. Cells were plated in 96-well plates (5×10^4 cells/well) and then treated with 20 μg/ml rhein. After treatment for 48 h, cell growth was examined using MTT assay. $OD_{570\text{-}630}$ in each well was measured with a micro-ELISA reader.
